# Aconitine accidental poisoning: two case reports and literature review

**DOI:** 10.1186/s12245-026-01339-2

**Published:** 2026-09-18

**Authors:** Qian Zhao, Lei Wang, Feng Zeng, Xue-bo Jiang, Yan-xia Shao

**Affiliations:** 1 Department of Emergency Medicine, The First Affiliated Hospital (Southwest Hospital) of Army Medical University (Third Military Medical University), Chongqing, China; 2 No. 29, Zhengjie Street, Gaotanyan, Shapingba District, Gaotanyan, Chongqing, 400030 People’s Republic of China

**Keywords:** Chinese herbal medicine, Aconitine poisoning, Arrhythmia, Hemoperfusion, Treatment

## Abstract

**Introduction:**

Chinese Herbal Medicine (CHM) is widely integrated into clinical practice across Asia and other regions for disease treatment and health maintenance. Aconitine, a potent diterpenoid alkaloid derived from plants of the Aconitum genus (e.g., *Aconitum carmichaelii* and *Aconitum kusnezoffii*), exhibits significant pharmacological activity but also considerable toxicity. While properly processed Aconitum herbs are prescribed for their analgesic and anti-rheumatic properties, inadequate detoxification during processing or improper clinical use can lead to accidental aconitine poisoning. This condition characterized by severe cardiotoxicity and neurotoxicity, which may progress to fatal outcomes. Recently, with the expanding clinical application of traditional medicines globally, the incidence of aconitine poisoning has risen, particularly in Asian populations.

**Case presentation:**

We present two cases of aconitine poisoning in middle-aged patients following ingestion of herbal preparations containing aconitine. Both patients developed significant cardiac arrhythmias of varying severity. Clinical management included gastric lavage, catharsis induced by mannitol, gastric mucosal protection, intravenous fluid resuscitation, and hemoperfusion. One patient experienced cardiac arrest and subsequently died after family members elected to withdraw life-sustaining therapy. The second patient was successfully weaned from mechanical ventilation and was eventually discharged following a short-term hospitalization.

**Conclusion:**

These cases emphasize the urgent need to enhance the awareness of healthcare professionals and the public regarding the risks of certain traditional Chinese medicines, especially those containing aconitine. Heightening vigilance and promoting their rational use are crucial for improving public health knowledge and preventing similar adverse events.

## Background

Aconitine, a highly toxic diterpenoid alkaloid from Aconitum species, is a leading cause of fatal herbal poisoning in Asia, with its use rising globally [1, 2]. Its narrow therapeutic index and the common practice of preparing raw plant material at home frequently lead to accidental intoxication, characterized by life-threatening ventricular dysrhythmias and cardiogenic shock [3]. Clinical diagnosis is challenging due to nonspecific presentations and the lack of rapid confirmatory testing in most emergency settings [4]. While numerous case reports exist, many describe management strategies that are discordant with current evidence-based toxicology [5, 6]. This report critically examines two contrasting cases of aconitine poisoning to highlight the gap between routine practice and international guidelines, proposing a practical, evidence-informed framework for diagnosis, management, and prevention.

## Case presentation

### Case 1

A 61-year-old female presented after ingesting a decoction prepared from the roots of Aconitum carmichaelii and Aconitum kusnezoffii on two occasions (at 13:00 and 19:00) on September 9, 2023; the dose administered on each occasion was not recorded. Approximately two hours after the second ingestion (at approximately 21:00), she developed dizziness, vomiting, palpitations, perioral and limb numbness, and generalized weakness. She was initially evaluated at a local traditional Chinese medicine hospital, where electrocardiography (ECG) revealed atrial fibrillation with third-degree atrioventricular block. She was subsequently transferred via emergency medical services to our department for further management, arriving at 04:37 the following day.

**Fig. 1:**
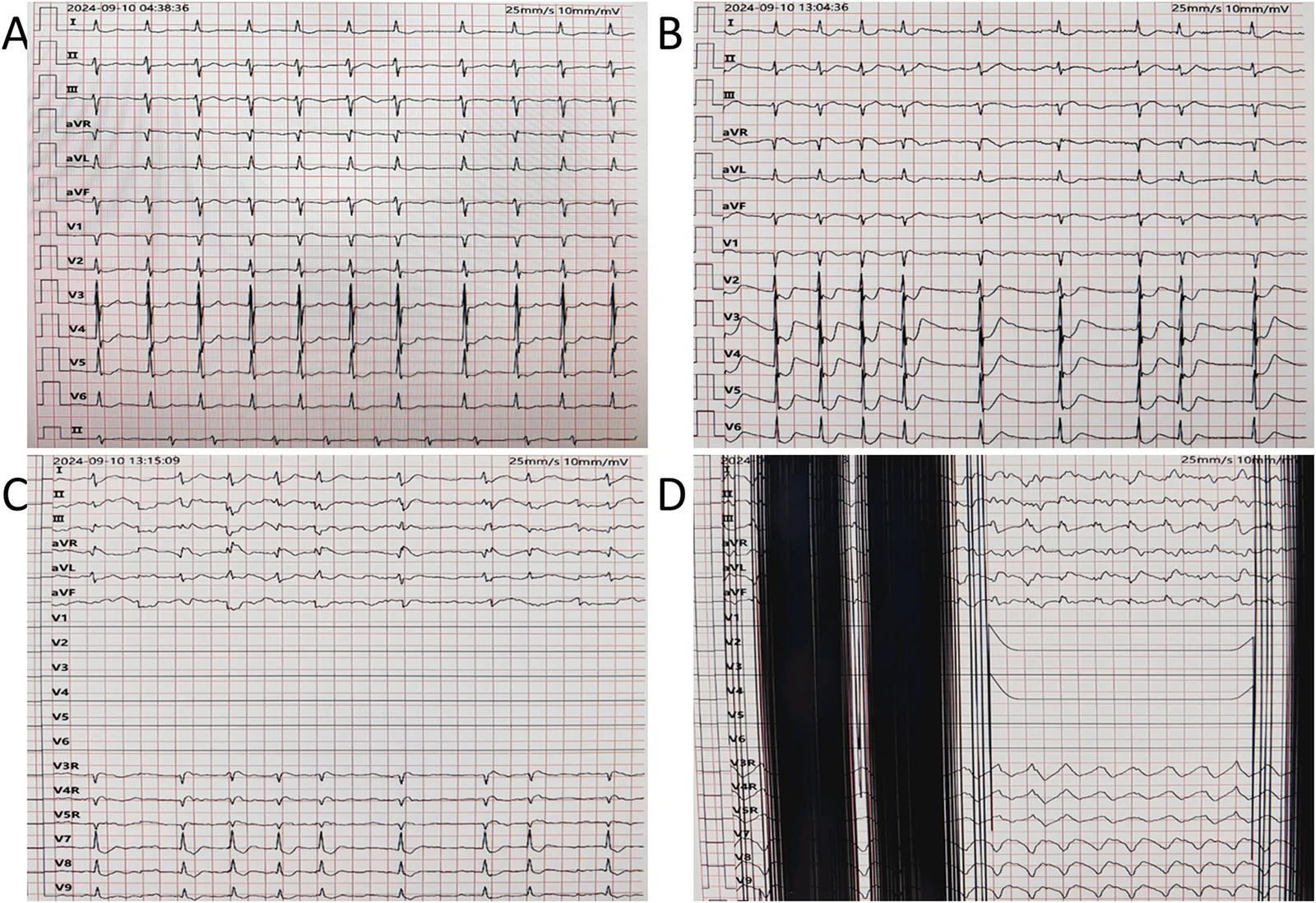
The changes in the electrocardiogram of patient 1

Physical examination on admission noted: body temperature, 36.5 °C; heart rate, 21 bpm; respiratory rate, 13 breaths/min; blood pressure, 76/36 mmHg, the patient was conscious but in apparent distress, with cold, clammy skin. Peripheral pulses were irregular and thready. Cardiac auscultation revealed an irregular rhythm with variable intensity of the first heart sound. A repeat ECG confirmed atrial fibrillation with ST-T segment changes and left axis deviation (Fig. 1A). Arterial blood gas analysis showed hyperkalemia and elevated lactate levels. Complete blood count, myocardial enzyme profiles, coagulation studies, and hepatic and renal function tests were within normal limits (Table 1).

**Table 1:** Case 1. Blood tests at hospital

Time	10.26	10.27	10.28	0.29
PH	7.29	7.41	7.35	7.42
PaCO2	55	29	34	34
PaO2 /FiO2	167	451	400	273
K	3.5	4.0	4.0	3.8
Lac	2.7	2.0	1.0	1.2
WBC*10^9^/L	7.54			5.94
Hb g/L	142			149
PLT*10^9^/L	205			84
Cr(umol/L)	136.15			58
APTT s	27.1	＞120	33.7	
PT s	11.5	17.3	12.5	
Fib g/L	2.7	2.61	2.88	
BNP pg/ml	654.48			
cTnI ng/ml	0.017			

Lac Lactate, WBC white blood cell, Hb hemoglobin, PLT platelet, Cr creatinine, APTT activated partial thromboplastin time, PT prothrombin time, Fib Fibrinogen, BNP brain natriuretic peptide, cTnI Troponin I

Following a diagnosis of aconitine poisoning, the patient underwent continuous ECG monitoring and received a series of detoxification measures, including gastric lavage, activated charcoal adsorption, and catharsis with mannitol. Supportive management included oxygen inhalation, intravenous fluid resuscitation, and anti-shock therapy, as well as potassium-lowering and diuretic agents. Atropine was administered for bradyarrhythmia. Hemoperfusion was initiated at 06:40. At 09:28, the patient developed recurrent bradycardia (HR 48 bpm), which was managed with additional atropine and isoproterenol infusion. By 13:04, the ECG showed atrial fibrillation (Fig. 1B), which was further confirmed with an 18-lead ECG at 13:15, demonstrating concomitant ST-T changes (Fig. 1A); fFollow-up laboratory evaluations were performed.

At 14:02, the patient felt palpitations with a heart rate of 181 bpm and blood pressure of 154/86 mmHg. ECG revealed ventricular tachycardia (Fig. 1C), prompting the lidocaine. At 14:04, she lost of consciousness with absence of palpable central pulses suddenly. The epinephrine and chest compressions were used Immediately. The arterial blood gas analysis and other laboratory tests were completed, and veno-arterial extracorporeal membrane oxygenation (VA-ECMO) was proposed as a rescue intervention. However, the family declined ECMO support for personal reasons. By 15:00, there was no return of spontaneous circulation, central pulses remained absent, and pupils were fixed and dilated with no light reflex. ECG demonstrated an equipotential line, and the patient was declared clinically deceased.

### Case 2

**Fig. 2:**
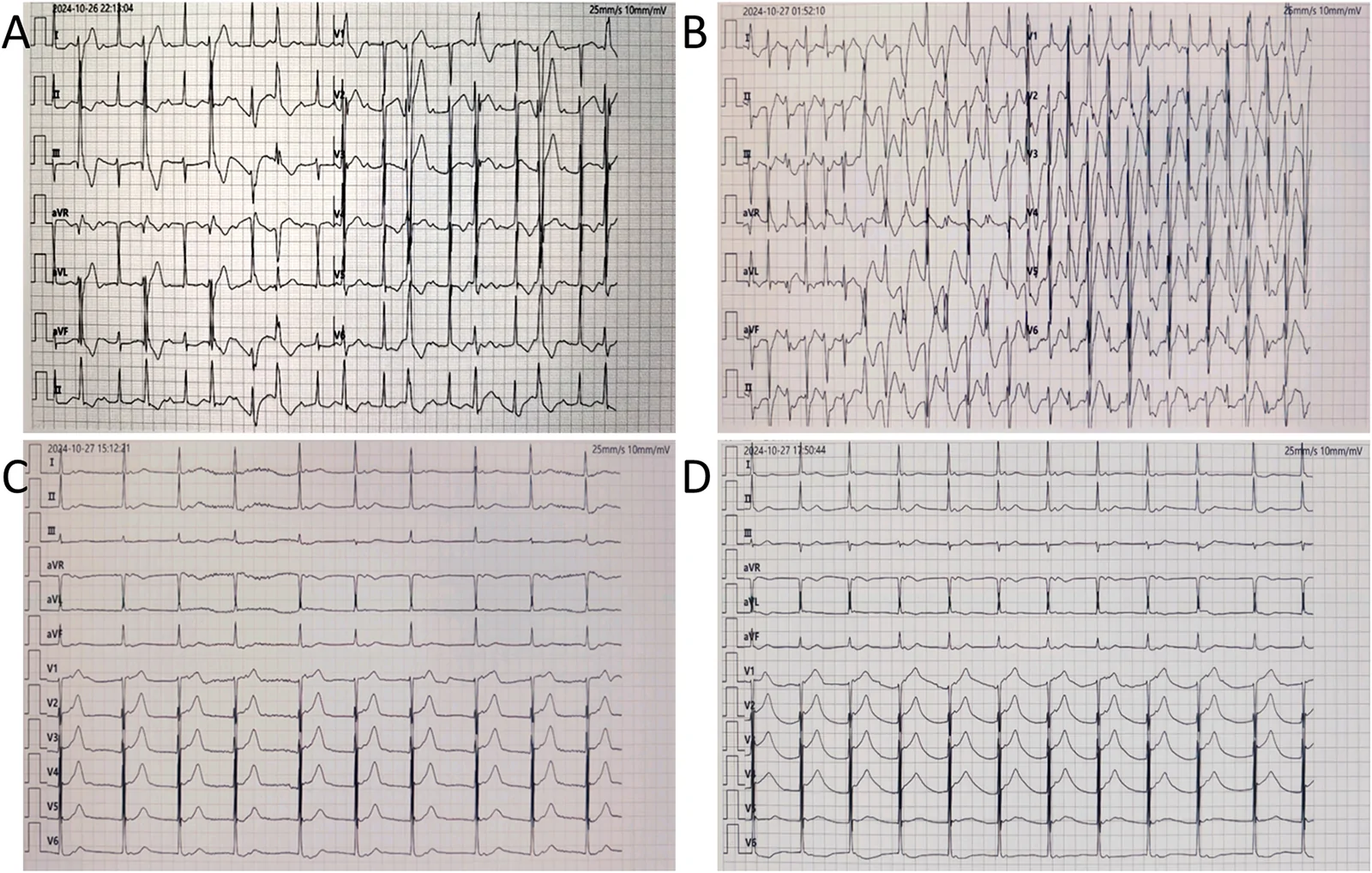
The changes in the electrocardiogram of patient 2

A 56-year-old male presented to the emergency department of our hospital at 21:00 on October 26, 2024, approximately two hours after ingesting wine prepared from unprocessed *Aconitum kusnezoffii*. Shortly after consumption, he developed neuropsychiatric symptoms, which progressed to gradual impairment of consciousness. An admission ECG revealed sinus tachycardia, frequent premature ventricular contractions, first-degree atrioventricular block, and ST-T segment abnormalities (Fig. 2A). Arterial blood gas analysis demonstrated hypercapnia and elevated lactate levels. While complete blood count, myocardial enzyme profiles, and coagulation parameters were within normal limits, hepatic and renal function tests showed mild abnormalities (Table 2).

**Table 2:** Case 2. Blood tests at hospital

Time	04:38	13:29	14:02
PH	7.35	7.38	7.38
PaCO2	36	37	58
PaO2 /FiO2	579	503	22
K	5.5	4.5	6.5
ca	1.05	1.07	1.09
Lac	2.5	1.8	6.6
HCO3	19.9	21.9	17.4
WBC *10^9^/L	12.5		
Hb g//L	126		
PLT*10^9^/L	209		
AST U/L	31.1		
ALT U/L	＜10		
TBIL umol/L	15.7		
Cr umol/L	75.68		
APTT s	22.2		
PT s	10.3		
Fib g/L	3.08		
BNP pg/ml	200.06	92.1	
cTnI ng/ml	＜0.012	0.03	
CK U/L	84.10		
CK-MB U/L	18.13	3.67	
Myo ng/ml	66.6	62.5	

Lac Lactate, WBC white blood cell, Hb hemoglobin, PLT platelet, Cr creatinine, AST aspartate aminotransferase, ALT alanine aminotransferase, APTT activated partial thromboplastin time, PT prothrombin time, Fib Fibrinogen, BNP brain natriuretic peptide, cTnI Troponin I,CK Creatine kinase, CK-MB Creatine kinase isoenzyme, Myo Myoglobin

A preliminary diagnosis of aconitine poisoning was established. The patient underwent emergent gastric lavage and endotracheal intubation for airway protection before being transferred to the emergency intensive care unit (EICU). His medical history was significant for hypertension, with previously recorded blood pressures up to 180/110 mmHg.

In the EICU, his course was complicated by recurrent ventricular arrhythmias. At 00:25 on October 27, continuous monitoring showed frequent PVCs with brief runs of ventricular tachycardia, managed with esmolol. At 00:50, he developed torsades de pointes (TdP) triggered by a PVC, which required immediate electrical defibrillation and intravenous magnesium sulfate, resulting in the restoration of sinus rhythm. Potassium and magnesium were supplemented for membrane stabilization. By 01:45, his heart rate increased to 150–180 bpm, with ECG confirming paroxysmal ventricular tachycardia (Fig. 2B). A cardiology consultation recommended aggressive detoxification, correction of ischemia and hypoxia, continued membrane stabilization, and consideration of electrical cardioversion or lidocaine infusion if warranted.

The patient’s treatment regimen included continued catharsis, activated charcoal, intravenous fluids, electrolyte repletion, and hemoperfusion. His vital signs, fluid balance, serial ECGs, and laboratory parameters were closely monitored (Fig. 2C). By hospital day 3, the patient’s condition markedly improved with stabilization of vital signs. Successful extubation was performed. On day 4, repeat complete blood count, hepatic and renal function, and coagulation profiles returned to normal ranges. The patient was discharged on day 5. Subsequent follow-up indicated complete recovery and return to normal activities.

Finally, we compared the conditions of the two cases, including the time of onset time after ingestion, estimated dose/preparation, ECG findings, treatments, and outcome (Table 3).

**Table 3:** Comparative clinical data of two aconitine poisoning patients

Items	Case 1 (61-year-old female)	Case 2 (56-year-old male)
Exposure route	wice oral aconitum decoction, unknown dosage	Single oral aconitum medicinal liquor, unknown dosage
Latency	Symptoms onset at 8 h post-ingestion	Symptoms onset within 2 h post-ingestion
ECG manifestations	AF + 3rd-degree AV block, progressive sustained VT, final asystole	Sinus tachycardia, frequent VPCs, 1st-degree AV block, recurrent non-sustained VT and torsades de pointes
Key treatments	Gastric lavage, activated charcoal, mannitol; atropine + isoproterenol; single hemoperfusion; lidocaine, CPR; VA-ECMO declined by family	Endotracheal intubation, lavage & catharsis, activated charcoal; esmolol, magnesium sulfate, polarizing fluid, electrical defibrillation; hemoperfusion; fluid & electrolyte support
Outcome	Refractory malignant arrhythmia, cardiac arrest, clinical death	Extubation on day 3, normal lab results on day 4, discharge on day 5, full recovery during follow-up

Abbreviations: AV, atrioventricular; ECG, electrocardiogram; PVCs, premature ventricular contractions; VA-ECMO, veno-arterial extracorporeal membrane oxygenation; VT, ventricular tachycardia; AF, Atrial fibrillation;CPR,Cardio-Pulmonary Resuscitation

## Discussion

The two cases presented herein provide a compelling natural experiment that underscores the critical determinants of outcome in severe aconitine poisoning: the timely application of mechanism-based therapies and the early deployment of mechanical circulatory support. Both patients developed refractory cardiac arrhythmias following the ingestion of aconitine-containing Chuanwu preparations, consistent with the toxin’s well-characterized pharmacodynamics [7, 8]. Aconitine exerts its lethal effects by persistently activating voltage-gated sodium channels, leading to delayed afterdepolarizations and re-entrant tachyarrhythmias [9, 10]. This mechanistic understanding is paramount, as it informs the choice of antiarrhythmic agents, favoring sodium-channel blockers (e.g., lidocaine, mexiletine) and amiodarone over standard ACLS algorithms, which are often ineffective [11]. The stark contrast in outcomes—fatal in Case 1 where veno-arterial extracorporeal membrane oxygenation (VA-ECMO) was declined, versus survival in Case 2 with supportive care—is the most powerful teaching point. Aconitine-induced cardiotoxicity is characteristically transient, with arrhythmias typically resolving within 24–48 h as the toxin is cleared (half-life ~ 3 h) [12, 13]. Therefore, the primary therapeutic goal is to provide a physiologic bridge through this critical window, and VA-ECMO has emerged as the decisive intervention for refractory ventricular dysrhythmias or cardiogenic shock [14, 15]. The failure to escalate care to mechanical support in the first case, in the setting of relentless arrhythmias and hemodynamic collapse, represents the single greatest missed therapeutic opportunity and should serve as a stark educational message for clinicians.

A critical appraisal of the specific interventions delivered in these cases against contemporary international toxicology guidelines reveals several practices that are obsolete, unsupported, or of unproven benefit. First, the use of gastric lavage hours post-ingestion is inconsistent with the position statements of the American Academy of Clinical Toxicology and the European Association of Poisons Centres and Clinical Toxicologists (AACT/EAPCCT), which restrict its use to a very narrow window (< 1 h) for life-threatening ingestions under airway protection [16, 17]. Given aconitine’s rapid absorption, the utility of lavage in these scenarios was negligible and potentially harmful. Similarly, the administration of mannitol as a cathartic has no established role in poisoning management and should be abandoned entirely [18]. While activated charcoal may offer benefit if given within the first hour, its use in these cases, given the delays, provided little to no benefit [19]. Second, the reliance on hemoperfusion as an established extracorporeal elimination therapy is not supported by robust pharmacokinetic evidence. Aconitine has a high volume of distribution and extensive tissue binding, rendering it poorly amenable to hemodialysis or hemoperfusion; these modalities should not be considered a cornerstone of management [20]. Third, the antiarrhythmic strategy requires substantial re-evaluation. The use of esmolol, a beta-blocker, lacks a mechanistic rationale in aconitine toxicity, which is driven by sodium channel activation. Conversely, the administration of magnesium for torsades de pointes in Case 2 was entirely appropriate and aligns with best practice [11]. Management must pivot to a mechanism-based approach, prioritizing lidocaine or amiodarone, which have been more consistently associated with successful rhythm restoration [11, 21]. This case series serves as a potent reminder that clinical toxicology practice must be anchored to pharmacokinetic and pharmacodynamic principles, moving away from reflex-driven interventions that offer no benefit and may delay definitive, life-saving care.

From these cases and the critical review above, we can distill a concise, stepwise management framework and a structured prevention strategy to fill a critical gap in the current literature. The first pillar is early recognition, based on a history of CHM exposure and characteristic ECG findings, including ventricular ectopy, bradyarrhythmias, and tachyarrhythmias [3]. Next, supportive care with airway protection and aggressive fluid resuscitation is fundamental. The third and most crucial pillar is mechanism-based antiarrhythmic therapy, utilizing sodium-channel blockers (lidocaine or mexiletine) and amiodarone, with magnesium reserved for torsades de pointes [11, 21]. The fourth pillar is early escalation to VA-ECMO for any patient with refractory dysrhythmias or cardiogenic shock, as this offers the only definitive bridge to survival [14, 22]. Finally, gastrointestinal decontamination with activated charcoal should be strictly limited to early (< 1 h), massive ingestions, in full concordance with AACT/EAPCCT guidelines [17–19]. Regarding prevention, the lessons from our cases, particularly the home-prepared medicinal wine in Case 2, are unequivocal. Public health initiatives must target the prohibition of home preparation of aconite-containing remedies and liquors, a primary source of severe poisoning [23]. This should be coupled with strict regulatory control over the sale of raw tubers, enforcement of preparation standards to ensure adequate detoxification, and clear, prominent labeling. The proven success of the public awareness campaign in Hong Kong, which led to a demonstrable reduction in incidence, provides direct evidence that such measures are effective [3, 24]. To support clinicians, we propose integrating these pillars into a practical algorithm, transforming this descriptive report into a potentially life-saving reference for the management of aconitine poisoning [25].

## Conclusion

Aconitine poisoning is a critical medical emergency with high mortality, primarily due to refractory ventricular arrhythmias and cardiogenic shock. This report demonstrates that favorable outcomes depend on early recognition, the swift application of mechanism-based antiarrhythmic therapy (sodium-channel blockers and amiodarone), and—most decisively—the timely escalation to VA-ECMO for patients with refractory dysrhythmias, as the toxicity is transient and survivable with adequate circulatory support. Conversely, routine use of gastric lavage, cathartics, and hemoperfusion is not supported by current evidence and should be abandoned. From a public health perspective, the prevention of aconitine poisoning requires targeted, evidence-informed measures: a ban on home preparation of aconite-containing remedies; strict regulation of raw tuber sales; enforcement of manufacturing standards for detoxification; and educational campaigns modeled on the successful Hong Kong initiative. Clinicians and policymakers must adopt this integrated, evidence-based approach to reduce the global burden of aconitine poisoning.

## Data Availability

No datasets were generated or analysed during the current study.
